# Provider & nursing perspectives on the “panculture”: opportunities for innovative diagnostic stewardship interventions

**DOI:** 10.1017/ash.2024.451

**Published:** 2024-11-11

**Authors:** Kevin M. Gibas, Leonard A. Mermel

**Affiliations:** 1 Department of Epidemiology & Infection Prevention, Rhode Island Hospital, Providence, RI, USA; 2 Department of Medicine, Warren Alpert Medical School of Brown University, Providence, RI, USA

## Abstract

**Objective:**

To examine practices of providers and nursing staff in evaluating febrile patients and identify drivers of excessive diagnostic testing.

**Design:**

Prospective multiple-choice surveys.

**Setting:**

Inpatient areas and the Emergency Department at Rhode Island Hospital (RIH) in Providence, RI.

**Participants & Methods:**

We conducted two surveys focused on the evaluation of febrile inpatients at RIH. One survey was of providers trained in internal medicine, surgery, pediatrics, emergency medicine, and neurology; the other survey was of nursing staff (registered nurses and certified nursing assistants), in inpatient areas and the emergency department.

**Results:**

70 providers (9%) and 178 nursing staff (12%) completed the surveys. 64% of providers (n = 43) reported “always” or “often” ordering full fever workups and 67% of providers (n = 47) reported “always” or “often” physically evaluating febrile patients. Nurses were less likely than providers to report that providers “always” or “often” physically evaluate febrile patients (n = 80, 45%; *P* < 0.01) and more likely to report providers “always” or “often” order full fever workups (n = 135, 76%; *P* = 0.04). 71% of providers (n = 50) reported “always” or “often” receiving written handoffs. 86% of providers (n = 60) reported handoffs are “always” or “often” accurate; however, only 17% of providers responded these were “always” accurate. 77% of providers (n = 54) reported “always” or “often” following handoff instructions to obtain a full fever workup for febrile patients, regardless of clinical status. Responses differed significantly by unit type and provider specialty and position.

**Conclusions:**

This study elucidates drivers of inefficient and excessive utilization of diagnostic studies and identifies targets for diagnostic stewardship interventions.

## Introduction

Fever in a hospitalized patient is a common clinical scenario with 2%–29% of hospitalized patients developing a fever during their hospitalization.^1,2^ There are guidelines addressing fever in critically ill patients, neutropenic patients and young children, but no guidelines addressing workup and management of febrile patients in general hospital units. The paucity of evidence and guidelines regarding the evaluation and management of febrile patients may contribute to the overutilization of diagnostic studies. Many providers order numerous tests and will often repeat diagnostic studies when a patient becomes febrile, irrespective of a patient’s symptoms or clinical status. This practice has become colloquially known as “panculturing,” conducting a “full fever workup,” or “culturing if spikes.”^3–5^ Providers often order extensive infectious workups and will often order these without physically evaluating patient at the bedside.^4,6–9^ Indiscriminately conducting broad diagnostic evaluations for fevers in hospitalized patients diverges from American College of Critical Care Medicine and Infectious Diseases Society of America’s recommendations that a new fever in a critically ill patient should trigger “a careful clinical assessment rather than automatic orders for laboratory and radiologic tests.”^10^

Factors driving the overutilization of diagnostic tests and excessive diagnostic evaluations in febrile inpatients include an overreliance on sign-out instructions, institutional culture, lack of transparency and uncertainty regarding costs of diagnostic tests, fear of malpractice, pressure from patients/families to order tests, not examining a patient to direct diagnostic testing, and a lack of peer/mentor role models practicing diagnostic stewardship.^2,4,11,12^ Practices for evaluating febrile patients also vary depending on a provider’s years of experience, the clinical service a patient is admitted to, the shift a provider is working, the location or unit where a patient is receiving care, and a provider’s role on the care team.^2,11^ These factors have the potential to drive overutilization of diagnostic tests, which has significant clinical implications including inappropriate and excessive prescription of antibiotics, increased hospital length of stay, increased healthcare costs, overdiagnosis of presumed healthcare-associated infections, and promotion of antimicrobial resistance.^9,13,14^ In addition, the overutilization of diagnostic tests and the resulting downstream effects have important implications for resource utilization, particularly given recent supply chain shortages of various medical supplies, such as blood culture bottles.

Providers recognize the diagnostic evaluations they order to evaluate febrile patients are often not evidence-based nor cost-effective.^3,11^ Despite this, when surveyed, more than 90% of physicians felt it is a responsibility of providers to limit unnecessary tests and to control healthcare costs.^12^ Given ongoing efforts to promote high-value care, improve health outcomes, improve resource utilization, and reduce healthcare costs, more data are necessary to elucidate individual and institution-specific drivers of excessive diagnostic testing and identify potential barriers to changing practices for evaluating febrile patients in hospital settings. We conducted a study examining the practices and perceptions of medical providers and nursing staff as it pertains to the evaluation of febrile patients in inpatient settings and the emergency department (ED) at Rhode Island Hospital (RIH). We hypothesized that providers would overestimate their frequency of physically evaluating febrile patients and that providers would often order full fever workups rather than conduct exam-directed testing in febrile patients.

## Methods

We conducted two prospective, multiple-choice surveys of clinical staff working in inpatient areas and the ED at RIH, a tertiary-care academic medical center licensed for 713 beds. One survey was distributed to medical providers including advanced practice providers (APPs) (nurse practitioners and physician assistants), house officers (residents and fellows), internal medicine and pediatric hospitalists, and other non-hospitalist staff physicians working in the following medical specialties: internal medicine or internal medicine subspecialities, surgery or surgical subspecialties, pediatrics or pediatrics subspecialities, emergency medicine, and neurology. The second survey was distributed to nursing staff including registered nurses (RNs) and certified nursing assistants (CNAs). The surveys were voluntary and anonymous, and each participant could only complete the survey once. Participants were sent a survey link and QR code linking to the survey using preexisting e-mail listservs and announcement platforms at RIH. Microsoft Forms was used to administer the survey and store data securely and anonymously. The surveys were conducted between January 3, 2024 and February 26, 2024. Two rounds of reminder e-mails to complete the survey were sent. This project met institutional review board criteria for exemption from formal review.

The surveys (Supplementary Figures 1 and 2) each consisted of 10 multiple-choice questions focusing on the practices and perceptions of medical providers and nursing staff as it pertains to evaluating febrile patients in the ED and inpatient settings. Questions asked in both the surveys of providers and the nursing staff addressed the frequency in which providers physically examine febrile patients at the bedside, how often a full fever work up (or “panculture”) is ordered when evaluating febrile patients, during which shifts a provider is most likely to order a full fever workup, and which diagnostic tests are most likely to be ordered to work up a febrile patient (Table 2). A full fever workup was defined as obtaining at least blood cultures and a urinalysis/urine culture plus one or more of the following tests: chest X-ray, respiratory pathogen panel, sputum culture, or *Clostridioides difficile* testing. These questions were developed using data from prior studies and input from the study investigators’ clinical experience and expertise in infectious diseases, hospital epidemiology, infection prevention, and internal medicine.^2,4,8,9,13,14^ The principal investigator who developed the survey questions has formal training in survey design, internal medicine, and infectious diseases.

**Table 1.** Participant characteristics

*Medical providers (n = 70)*	
Characteristic	Number (% of Total)
Type of Provider	
*Advanced Practice Provider (APP)*	8 (11.4%)
*House officer (Residents & Fellows)*	44 (62.9%)
*Hospitalist*	14 (20.0%)
*Non-Hospitalist Staff Physician*	4 (5.7%)
Specialty	
*Internal Medicine or Internal Medicine subspeciality*	27 (38.6%)
*Surgical specialty or Surgical subspeciality*	15 (21.4%)
*Pediatrics or Pediatrics subspeciality*	22 (31.4%)
*Neurology*	1 (1.4%)
*Emergency Medicine*	5 (7.1%)
*Nursing Staff (N = 178)*	
Nursing Certification	
*Registered Nurse (RN)*	168 (94.4%)
*Certified Nursing Assistant (CNA)*	10 (5.6%)
Primary Work Area/Unit	
*Adult Medical-Surgical (Med-Surg) Unit/Floor*	55 (30.1%)
*Adult ICU & Step-down Unit/Floor*	55 (30.1%)
*Pediatric Medical-Surgical (Med-Surg) Unit/Floor*	28 (15.7%)
*Pediatric ICU & Step-down Unit/Floor*	10 (5.6%)
*Oncology or Stem Cell Transplant Unit/Floor*	19 (10.7%)
Other Inpatient Unit *	11 (6.2%)
Primary Shift	
*Day Shift*	95 (53.4%)
*Second Shift*	23 (12.9%)
*Night/Overnight Shift*	60 (33.7%)

* Includes the emergency department, postanesthesia acute care unit (PACU), behavioral health units.

**Table 2.** Survey responses^1^

	Medical providers (n = 70)	Nursing staff (n = 178)	^2^
How often does the provider evaluate a febrile patient in person? *(“Provider evaluates in person”)* ^3^			
*Always*	14 (20.0%)	16 (9.0%)	*P* < 0.01
*Often*	33 (47.1%)	64 (36.0%)	
*Rarely*	23 (32.9%)	86 (48.3%)	
*Never*	0 (0.0%)	12 (6.7%)	
How often is a full fever work up (“panculture”) ordered for a febrile patient? * *(“Full fever workup ordered?”)*			
*Always*	5 (7.1%)	20 (11.2%)	*P* = 0.04
*Often*	38 (54.3%)	115 (64.6%)	
*Rarely*	24 (34.3%)	42 (23.6%)	
*Never*	3 (4.3%)	1 (0.6%)	
When is a provider more likely to order a full fever work up (“panculture”)? *(“Most likely shift to order full fever work up”)*			
*The shift does not matter*	56 (80.0%)	122 68.5%)	*P* < 0.01
*Day shift*	2 (2.9%)	48 (27.0%)	
*Night shift*	12 (17.1%)	8 (4.5%)	
*Weekend shift*	0 (0.0%)	0 (0.0%)	
When a full fever work up (“panculture”) is ordered, what tests are generally ordered?			
*Blood culture*	70 (100%)	166 (93.3%)	
*Urine culture*	66 (94.3%)	137 (77.0%)	
*C. diff testing*	2 (2.9%)	8 (4.5%)	
*Sputum culture*	10 (14.3%)	37 (20.8%)	
*Chest X-ray*	58 (82.9%)	108 (60.7%)	
*Respiratory pathogen panel*	54 (77.1%)	106 (59.6%)	
Medical Provider Specific Questions			
You are more likely to order a full fever work up (“panculture”) if you:			
*Go to the unit to examine the patient*	11 (15.7%)		
*Do not go to the unit to examine the patient.*	7 (10.0%)		
*It does not matter if you (the provider) go to the unit to examine the patient or not.*	52 (74.3%)		
When assuming care of a patient how often do you receive a written handoff for that patient? (*“Receive handoff”*)			
*Always*	39 (55.7%)		
*Often*	11 (15.7%)		
*Rarely*	11 (15.7%)		
*Never*	9 (12.9%)		
If the handoff contains instructions to order a full fever work up (“panculture”), how often do you follow these instructions regardless of the patient’s clinical status? (*“Follow handoff instructions”)*			
*Always*	28 (40.0%)		
*Often*	26 (37.1%)		
*Rarely*	9 (12.9%)		
*Never*	8 (10.0%)		
How often do you feel that the handoffs/sign-outs you receive are accurate and up to date? (“*Handoffs accurate/up to date*”)			
*Always*	12 (17.1%)		
*Often*	48 (68.6%)		
*Rarely*	10 (14.3%)		
*Never*	0 (0.0%)		
Nursing Staff Specific Question			
When a patient you are caring for is febrile, how often do you notify the provider on call?			
*Always*		141 (79.2%)	
*Often*		31 (17.4%)	
*Rarely*		1 (0.6%)	
*Never*		5 (2.8%)	
When a provider orders a full fever work up (“panculture”), how often do they evaluate the patient in person? (“*Evaluate in person*”)			
*Always*		36 (20.2%)	
*Often*		62 (34.8%)	
*Rarely*		72 (40.5%)	
*Never*		8 (4.5%)	

1 A full fever workup was defined as obtaining at least blood and a urinalysis/urine culture plus any of the following: chest X-ray, respiratory pathogen panel, sputum culture, or *Clostridioides difficile* (*C. difficile*) testing.

2 For questions common to both surveys for medical providers and nursing staff, differences in responses between providers and nursing staff were described using a chi-square test (χ2) or Fisher’s exact test.

3 For questions that have a phrase in parentheses after them, that phrase will be the abbreviated text for that question in subsequent tables (Tables 3 and 4).

Survey data was analyzed descriptively for all categorical variables. Regarding questions common to both surveys for medical providers and nursing staff, differences in responses between providers and nursing staff were described using a chi-square test (χ2) or Fisher’s exact test (Table 2). Differences in nursing staff responses by unit type, provider responses by medical specialty, and provider responses by position were also described using a χ2 or Fisher’s exact test (Tables 3 and 4). Fischer’s exact test was used when >20% of expected cell counts were less than five observations. χ2 was used when ≤20% of expected cell counts were less than five observations.^15,16^ Analyses were conducted using STATA 18.0 (Stata Corp).^17^

**Table 3.** Nursing staff responses by unit type


	Adult medical-surgical (N = 55)	Adult ICU & step-down (n = 55)	Pediatric medical-surgical (n = 28)	Pediatric ICU & step-down (n = 10)	Oncology & transplant (n = 19)	Other (n = 11)	
Provider evaluates in person?							
*Always*	4 (7.3%)	10 (18.2%)	0 (0.0%)	1 (10.0%)	1 (5.3%)	0 (0.0%)	P < 0.01
*Often*	13 (23.6%)	18 (32.7%)	16 (57.1%)	7 (70.0%)	4 (21.1%)	6 (54.6%)	
*Rarely*	32 (58.2%)	26 (47.3%)	11 (39.3%)	2 (20.0%)	11 (57.9%)	4 (36.4%)	
*Never*	6 (10.9%)	1 (1.8%)	1 (3.6%)	0 (0.0%)	3 (15.8%)	1 (9.1%)	
Full fever workup ordered?							
*Always*	7 (12.7%)	10 (18.2%)	0 (0.0%)	0 (0.0%)	1 (5.3%)	2 (18.2%)	P < 0.01
*Often*	37 (67.3%)	41 (74.6%)	14 (50.0%)	4 (40.0%)	14 (73.7%)	5 (45.4%)	
*Rarely*	11 (20.0%)	4 (7.3%)	13 (46.4%)	6 (60.0%)	4 (21.0%)	4 (36.4%)	
*Never*	0 (0.0%)	0 (0.0%)	1 (3.6%)	0 (0.0%)	0 (0.0%)	0 (0.0%)	
Most likely shift to order full fever work up?							
*Does not impact*	33 (60.0%)	38 (69.1%)	24 (85.7%)	8 (80.0%)	10 (52.6%)	9 (81.8%)	P = 0.31
*Day shift*	4 (7.3%)	2 (3.6%)	0 (0.0%)	0 (0.0%)	1 (5.3%)	1 (9.1%)	
*Night shift*	18 (32.7%)	15 (27.3%)	4 (14.3%)	2 (20.0%)	8 (42.1%)	1 (9.1%)	
*Weekend shift*	0 (0.0%)	0 (0.0%)	0 (0.0%)	0 (0.0%)	0 (0.0%)	0 (0.0%)	

**Table 4.** Provider responses

Provider responses by specialty						
	Internal medicine (n = 27)	Pediatrics (n = 22)	Surgery (n = 15)	Emergency medicine (n = 5)	Neurology (n = 1)	
Provider evaluates in person?						
*Always*	4 (14.8%)	5 (22.7%)	4 (26.7%)	1 (20.0%)	0 (0.0%)	P = 0.44*
*Often*	11 (40.7%)	10 (45.5%)	8 (53.3%)	4 (80.0%)	0 (0.0%)	
*Rarely*	12 (44.4%)	7 (31.8%)	3 (20.0%)	0 (0.0%)	1 (100.0%)	
*Never*	0 (0.0%)	0 (0.0%)	0 (0.0%)	0 (0.0%)	0 (0.0%)	
Full fever workup ordered?						
*Always*	2 (7.4%)	0 (0.0%)	2 (13.3%)	0 (0.0%)	1 (100.0%)	P < 0.01
*Often*	16 (59.3%)	6 (27.3%)	11 (73.3%)	5 (100.0%)	0 (0.0%)	
*Rarely*	7 (25.9%)	15 (68.2%)	2 (13.3%)	0 (0.0%)	0 (0.0%)	
*Never*	2 (7.4%)	1 (4.6%)	0 (0.0%)	0 (0.0%)	0 (0.0%)	
Most likely shift to order full fever work up?						
*Does not impact*	19 (70.4%)	20 (90.9%)	11 (73.3%)	5 (100.0%)	1 (100.0%)	P = 0.36
*Days shift*	7 (25.9%)	1 (4.6%)	4 (26.67%)	0 (0.0%)	0 (0.0%)	
*Night shift*	1 (3.7%)	1 (4.6%)	0 (0.0%)	0 (0.0%)	0 (0.0%)	
*Weekend shift*	2 (7.4%)	1 (4.6%)	0 (0.0%)	0 (0.0%)	0 (0.0%)	
Receive handoff?						
*Always*	10 (37.0%)	16 (72.7%)	10 (66.7%)	3 (60.0%)	0 (0.0%)	P = 0.03
*Often*	7 (25.9%)	1 (4.6%)	2 (13.3%)	0 (0.0%)	1 (100.0%)	
*Rarely*	8 (29.6%)	1 (4.6%)	2 (13.3%)	0 (0.0%)	0 (0.0%)	
*Never*	2 (7.4%)	4 (18.2%)	1 (6.7%)	2 (40.0%)	0 (0.0%)	
Follow handoff instructions?						
*Always*	12 (44.4%)	5 (22.7%)	8 (53.3%)	2 (40.0%)	1 (100.0%)	P = 0.67
*Often*	8 (29.6%)	11 (50.0%)	4 (26.7%)	3 (60.0%)	0 (0.0%)	
*Rarely*	5 (18.5%)	3 (13.6%)	1 (6.7%)	0 (0.0%)	0 (0.0%)	
*Never*	2 (7.4%)	3 (13.6%)	2 (13.3%)	0 (0.0%)	0 (0.0%)	
Handoffs accurate/up to date?						
*Always*	6 (22.2%)	5 (22.7%)	0 (0.0%)	0 (0.0%)	1 (100.0%)	P = 0.10
*Often*	15 (55.6%)	16 (72.7%)	13 (86.7%)	4 (80.0%)	0 (0.0%)	
*Rarely*	6 (22.2%)	1 (4.6%)	2 (13.3%)	1 (20.0%)	0 (0.0%)	
*Never*	0 (0.0%)	0 (0.0%)	0 (0.0%)	0 (0.0%)	0 (0.0%)	

Provider Responses by Position						
	House Officer (n = 44)	Hospitalist (n = 14)	APP (n = 8)	Non-Hospitalist Staff Physician (n = 5)	Neurology (n = 1)	
Provider evaluates in person?						
*Always*	8 (18.2%)	4 (28.6%)	2 (25.1)	0 (0.0%)		P = 0.36
*Often*	24 (54.6%)	3 (21.4%)	4 (50.0%)	2 (50.0%)		
*Rarely*	12 (27.3%)	7 (50.0%)	2 (25.1)	2 (50.0%)		
*Never*	0 (0.0%)	0 (0.0%)	0 (0.0%)	0 (0.0%)		
Full fever workup ordered?						
*Always*	2 (4.6%)	1 (7.1%)	2 (25.1)	0 (0.0%)		P = 0.25
*Often*	26 (59.1%)	5 (35.7%)	5 (62.5%)	2 (50.0%)		
*Rarely*	15 (34.1%)	6 (42.9%)	1 (12.5%)	2 (50.0%)		
*Never*	1 (2.3%)	2 (14.3%)	0 (0.0%)	0 (0.0%)		
Most likely shift to order full fever work up?						
*Does not impact*	36 (81.8%)	11 (78.6%)	6 (75.0%)	3 (75.0%)		P = 0.78
*Days shift*	7 (15.9%)	2 (14.3%)	2 (25.0%)	1 (25.0%)		
*Night shift*	1 (2.3%)	1 (7.1%)	0 (0.0%)	0 (0.0%)		
*Weekend shift*	0 (0.0%)	0 (0.0%)	0 (0.0%)	0 (0.0%)		
Receive handoff?						
*Always*	35 (79.6%)	4 (28.6%)	0 (0.0%)	0 (0.0%)		P < 0.01
*Often*	3 (6.8%)	5 (35.7%)	2 (25.0%)	1 (25.0%)		
*Rarely*	4 (9.1%)	3 (21.4%)	4 (50.0%)	0 (0.0%)		
*Never*	2 (4.6%)	2 (14.3%)	2 (25.0%)	3 (75.0%)		
Follow handoff instructions?						
*Always*	35 (79.6%)	4 (28.6%)	0 (0.0%)	0 (0.0%)		P = 0.02
*Often*	3 (6.8%)	5 (35.7%)	2 (25.0%)	1 (25.0%)		
*Rarely*	4 (9.1%)	3 (21.4%)	4 (50.0%)	0 (0.0%)		
*Never*	2 (4.6%)	2 (14.3%)	2 (25.0%)	3 (75.0%)		
Handoffs accurate/up to date?						
*Always*	4 (9.1%)	5 (35.7%)	1 (12.5%)	2 (50.0%)		P = 0.01
*Often*	36 (81.8%)	7 (50.0%)	4 (50.0%)	1 (25.0%)		
*Rarely*	4 (9.1%)	2 (14.3%)	3 (37.5%)	1 (25.0%)		
*Never*	0 (0.0%)	0 (0.0%)	0 (0.0%)	0 (0.0%)		

## Results

Seventy of 777 (9.0%) medical providers, 168 of 1066 (16%) registered nurses (RNs), and 10 of 456 (2.2%) nursing assistants (CNAs) working in inpatient areas and the ED completed the surveys (Table 1). Of the providers, 44 (63%) were house officers, 14 (20%) were hospitalists, 4 (6%) were non-hospitalist staff physicians, and 8 (11%) were APPs. Twenty-seven (39%) providers were trained in internal medicine or an internal medicine subspeciality while 22 (31%) were trained in pediatrics or a pediatrics subspeciality, 15 (21%) in surgery or a surgical subspeciality, 5 (7.1%) in emergency medicine, and 1 (1.4%) in neurology. Of the nursing staff who completed the survey, 55 (30%) primarily worked in an adult medical-surgical unit, 55 (30%) worked in an adult intensive care unit (ICU) or adult step-down unit, 28 (16%) worked in a pediatric medical-surgical unit, 10 (5.6%) worked in a pediatric ICU or step-down unit, 19 (11%) worked on an oncology or stem cell transplant unit, and 11 (6.2%) worked on another type of unit.

Over two-thirds of providers reported “always” or “often” ordering full fever workups when evaluating febrile patients; however, this varied significantly by medical specialty (Table 4). All providers trained in emergency medicine and neurology, as well as a majority of surgical and internal medicine providers, reported “always” or “often” ordering full fever workups when evaluating febrile patients. In contrast, only six pediatric providers (27%) responded they “always” or “often” obtain a full fever workup when evaluating febrile patients. There was no significant difference in how often providers reported ordering full fever workups by their position.

Over three quarters of nursing staff surveyed responded providers “always” or “often” order a full fever workup (Table 2). Nursing staff working on adult medical-surgical units and on oncology and stem cell transplant units were the most likely to report that full fever workups are routinely ordered to evaluate febrile patients (Table 3)..There were no significant differences in reported ordering practices based on shift by either specialty or provider position.

When asked how often they physically examine febrile patients at the bedside, approximately two-thirds of providers responded “always” or “often” (Table 2). There was no statistically significant difference in how often providers reported evaluating patients at the bedside by medical specialty or position (Table 4). In contrast, a majority of nursing staff (n = 98, 55%) responded that providers “rarely” or “never examine febrile patients at the bedside. Nursing staff working on oncology and stem cell transplant units were most likely to report that febrile patients are “always” or “often” evaluated by providers in person (Table 3). In contrast, nursing staff working on adult medical-surgical units were least likely to report that febrile patients are routinely examined by providers with less than one-third of nursing staff on these units reporting that providers “always” or “often” examine febrile patients at the bedside.

When asked about handoff instructions, a majority of providers reported “always” or “often” receiving written handoff instructions when assuming care of a patient (Table 2). The frequency in which providers reported receiving handoff instructions varied significantly by provider specialty and position (Table 4). Providers trained in pediatrics were most likely to report “always” receiving written handoff instructions, while internal medicine providers were least likely to report “always” receiving written handoff instructions (Table 4). When evaluating handoff practices by provider position, house officers were the most likely group to report “always” or “often” receiving written handoffs when assuming care of patients while APPs and non-hospitalist staff physicians were the least likely to report “always” or “often” receiving written handoffs. A majority of providers reported that the handoff instructions they receive are “always” or “often” accurate and up to date; however, of these, only 12 providers (17%) reported that they felt the sign-out instructions are “always” accurate and up to date (Table 2). House officers were the group most likely to report handoffs are accurate and up to date with greater than90% of house officers reporting their handoffs “always” or “often” accurate and up to date (Table 4). There was no significant difference in reported perceptions of handoff accuracy between provider specialties.

Providers were also asked how often they follow handoff instructions to obtain a full fever workup if a patient becomes febrile, regardless of the patient’s clinical status or prior workup. Over three quarters of providers reported “always” or “often” following the handoff instructions regardless of the patient’s clinical status. House officers were significantly more likely than other providers to report “always” or “often” following handoff instructions to order a full fever workup regardless of a patient’s clinical status (Table 4). There was no significant difference in reported practices for following handoff instructions by provider specialty.

## Conclusion/discussion

In this study, we surveyed medical providers to gauge their perspective regarding how often they examine patients when called by nursing staff about a febrile patient. We hypothesized that providers would overestimate the frequency of doing so and replace a sign or symptom-driven workup by conducting a full fever work up or “panculture.” We also surveyed nursing staff to determine the difference between the perception of medical staff responding that they always or often examine patients and the recollection of nurses regarding this issue. Our findings suggest that full fever workups are often ordered to evaluate febrile patients without directly asking the patient about their symptoms, nor physically examining the patient. Although approximately two-thirds of providers reported routinely physically assessing febrile patients in-person, when nursing staff were surveyed, over half reported that providers rarely or never evaluate febrile patients at the bedside. These findings are also consistent with prior studies which found that providers often order full fever workups without physically evaluating patients.^3,4^ This is critical as conducting a bedside evaluation and examination may provide important insights into the source of a patient’s fever and help target additional diagnostic testing. The importance of physically evaluating patients is highlighted by data from a study comparing outcomes with bedside versus telephone evaluations for the management of *Staphylococcus aureus* bacteremia which found that patients with a bedside consultation were more likely to have a deep focus of infection identified and had lower mortality than patients with telephone consultation at seven, 28, and 90 days.^18^

Our findings suggest that overreliance on handoff instructions, which many providers felt were not always accurate or up to date, may drive overutilization of unnecessary diagnostic studies when working up fevers in the hospital setting. Approximately 56% of providers reported routinely receiving written handoff instructions when assuming care of patients; however, this varied significantly by provider specialty and position. Over three quarters of respondents reported following handoff instructions to obtain a full fever workup if the patient became febrile, regardless of the patient’s clinical status or symptoms. These results echo prior studies which have shown that providers often report relying on handoff instructions but question their accuracy—including one study that found medical residents were 16 times more likely to order blood cultures on patients with handoff instructions to perform a full fever workup.^3,4^ To improve the quality and efficiency of patient care, healthcare institutions should promote handoff practices that encourage providers to perform careful individualized clinical assessments rather than the one-size-fits-all approach of conducting broad diagnostic evaluations, such as the full fever workup.

Our findings suggest that the practice of panculturing patients is widespread, often not directed at a patient’s signs or symptoms.^2–4,8,9,11,13,19^ Based on our review of the literature, this practice is not unique to our institution. Such overutilization of diagnostic studies can result in inappropriate and excessive antibiotic use, missed diagnoses, increased hospital length of stay, increased healthcare costs, challenges with resource allocation, lost hospital revenue, overdiagnosis of presumed healthcare-associated infections, and promotion of antimicrobial resistance.^9,13,14,20^ To address these issues and promote high-value care, it is imperative for healthcare institutions to adopt diagnostic stewardship programs that target the underlying individual and institutional drivers of inappropriate diagnostic testing, work toward strategic reductions in inappropriate testing, and optimize the process of ordering, performing, and reporting of diagnostic studies.^21^ Such programs should include provider education focused on both appropriate diagnostic testing and the potential adverse consequences of ordering unnecessary diagnostic studies.^22^ To ensure the success and sustainability of these programs, it will be essential for healthcare institutions to invest in the infrastructure, resources, and staffing to implement and monitor diagnostic stewardship interventions and to develop standardized measures to track excessive and inappropriate diagnostic testing.^23^

This study is subject to certain limitations. First, the surveys were conducted at a single academic medical center and had relatively low response rates, particularly among providers, which may limit the generalizability of these results beyond our institution or like institutions. The low response rate subjects these surveys to possible both response and non-response bias, as well as ascertainment bias which may limit the internal validity of these results. In addition, given the low response rates, nursing staff completing the survey may not have been observing the same patients or situations as the providers completing the survey making it difficult to directly compare data between the two groups. Additionally, this study would not capture specific situations in which nursing staff may not have recognized that a provider evaluated patient. This study may also not capture data from situations in which it may be appropriate for diagnostic tests to be ordered without immediately evaluating the patient at the bedside, such as when respiratory virus testing is ordered to identify patients who may need to be placed on appropriate isolation precautions, when a there is a clinical situation in which a provider expects a patient to have a fever and has already evaluated the patient recently, or when a provider has recently and thoroughly examined a patient and, as a result, do not feel the need to make a separate visit when notified about a fever. Another limitation of this study is that it did not collect patient-specific data, such as the acuity of the patient’s clinical status, which may influence how likely providers are to order diagnostic studies, evaluate a patient in person, or rely on handoff instructions for guidance. Given subject matter of the questions, this study may have been subject to social desirability bias where respondents were more likely to answer the questions based on how they thought they were expected to answer rather than based on their actual practices. To limit the potential impact of this, the survey was made anonymous and voluntary. Given this survey was voluntary, this study is also potentially subject to non-response bias. Finally, the month prior to conducting this study, our institution implemented a diagnostic stewardship program focused on reducing the ordering of unnecessary urine cultures. This program included a new urinalysis/urine culture order in our electronic medical record where urinalyses only reflex to a urine culture if certain criteria are met. Given this new urine culture diagnostic stewardship program, the new urinalysis/urine culture order, and education that providers and nursing received about this intervention around the time of these surveys were being conducted, providers and nurses may have been more acutely aware of the importance of diagnostic stewardship and best practices for ordering cultures, which may have influenced their practices and survey responses.

In sum, the practice of broad diagnostic testing (ie, panculture) of febrile patients that is not based on presenting signs or symptoms is common. There is a need for reaffirming the importance of physically assessing febrile, hospitalized patients to determine the best diagnostic approach and subsequent management plan.

## Supporting information

Gibas and Mermel supplementary material 1 Gibas and Mermel supplementary material

Gibas and Mermel supplementary material 2 Gibas and Mermel supplementary material

## Supplementary material

The supplementary material for this article can be found at https://doi.org/10.1017/ash.2024.451.

## Research transparency and reproducibility

The data that support the findings of this study are available from the corresponding author, Kevin M. Gibas, upon reasonable request.

## References

[1] Kaul DR , Flanders SA , Beck JM , Saint S. Incidence, etiology, risk factors, and outcome of hospital-acquired fever. J Gen Intern Med 2006;21:1184–1187. doi: 10.1111/j.1525-1497.2006.00566.x 17026728 PMC1831668

[2] Foong KS , Munigala S , Kern-Allely S , Warren DK. Blood culture utilization practices among febrile and/or hypothermic inpatients. BMC Infect Dis 2022;22:779. doi: 10.1186/s12879-022-07748-x 36217111 PMC9552399

[3] Howard-Anderson J , Schwab K , Quinn R , Graber CJ. Choosing Wisely Overnight? Residents’ Approach to Fever. Open Forum Infect Dis 2017;4:ofx080. doi: 10.1093/ofid/ofx080 28638842 PMC5473033

[4] Howard-Anderson J , Schwab KE , Chang S , Wilhalme H , Graber CJ , Quinn R. Internal medicine residents’ evaluation of fevers overnight. Diagn Berl Ger 2019;6:157–163. doi: 10.1515/dx-2018-0066 PMC6541517 30875319

[5] Gutow AP. The use of blood cultures. JAMA 1993;269:3109–3110. 8505811

[6] Linsenmeyer K , Gupta K , Strymish JM , Dhanani M , Brecher SM , Breu AC. Culture if spikes? Indications and yield of blood cultures in hospitalized medical patients. J Hosp Med 2016;11:336–340. doi: 10.1002/jhm.2541 26762577

[7] Lesperance R , Lehman R , Lesperance K , Cronk D , Martin M. Early postoperative fever and the “routine” fever work-up: results of a prospective study. J Surg Res 2011;171:245–250. doi: 10.1016/j.jss.2010.03.009 20655062

[8] Golob JF , Claridge JA , Sando MJ , et al. Fever and leukocytosis in critically ill trauma patients: it’s not the urine. Surg Infect 2008;9:49–56. doi: 10.1089/sur.2007.023 18363468

[9] Albin OR , Saravolatz L , Petrie J , Henig O , Kaye KS. Rethinking the “pan-culture”: clinical impact of respiratory culturing in patients with low pretest probability of ventilator-associated pneumonia. Open Forum Infect Dis 2022;9:ofac183. doi: 10.1093/ofid/ofac183 35774933 PMC9239552

[10] O’Grady NP , Barie PS , Bartlett JG , et al. Guidelines for evaluation of new fever in critically ill adult patients: 2008 update from the American college of critical care medicine and the infectious diseases society of America. Crit Care Med 2008;36:1330–1349. doi: 10.1097/CCM.0b013e318169eda9 18379262

[11] Sedrak MS , Patel MS , Ziemba JB , et al. Residents’ self-report on why they order perceived unnecessary inpatient laboratory tests. J Hosp Med 2016;11:869–872. doi: 10.1002/jhm.2645 27520384

[12] Colla CH , Kinsella EA , Morden NE , Meyers DJ , Rosenthal MB , Sequist TD. Physician perceptions of choosing wisely and drivers of overuse. Am J Manag Care 2016;22:337–343. 27266435

[13] Chen AI , Bilker WB , Hamilton KW , O’Donnell JA , Nachamkin I. Blood culture utilization at an academic hospital: Addressing a gap in benchmarking. Infect Control Hosp Epidemiol 2018;39:1353–1359. doi: 10.1017/ice.2018.231 30261936

[14] Advani SD , Gao CA , Datta R , et al. Knowledge and practices of physicians and nurses related to urine cultures in catheterized patients: an assessment of adherence to IDSA guidelines. Open Forum Infect Dis 2019;6:ofz305. doi: 10.1093/ofid/ofz305 31375836 PMC6677670

[15] The Chi squared tests | The BMJ. The BMJ | The BMJ: leading general medical journal. Research. Education. Comment. Published October 28, 2020. https://www.bmj.com/about-bmj/resources-readers/publications/statistics-square-one/8-chi-squared-tests. Accessed March 13, 2024.

[16] Bewick V , Cheek L , Ball J. Statistics review 8: qualitative data - tests of association. Crit Care Lond Engl 2004;8:46–53. doi: 10.1186/cc2428 PMC420070 14975045

[17] New in Stata 18 | Stata. https://www.stata.com/new-in-stata/. Accessed March 13, 2024.

[18] Forsblom E , Ruotsalainen E , Ollgren J , Järvinen A. Telephone consultation cannot replace bedside infectious disease consultation in the management of *Staphylococcus aureus* Bacteremia. Clin Infect Dis Off Publ Infect Dis Soc Am 2013;56:527–535. doi: 10.1093/cid/cis889 23087397

[19] Fanning J , Neuhoff RA , Brewer JE , Castaneda T , Marcotte MP , Jacobson RL. Frequency and yield of postoperative fever evaluation. Infect Dis Obstet Gynecol 1998;6:252–255. doi: 10.1002/(SICI)1098-0997(1998)6:6<252::AID-IDOG6>3.0.CO;2-4 9972487 PMC1784817

[20] Morgan DJ , Malani PN , Diekema DJ. Diagnostic stewardship to prevent diagnostic error. JAMA 2023;329:1255–1256. doi: 10.1001/jama.2023.1678 36862424

[21] Fabre V , Davis A , Diekema DJ , et al. Principles of diagnostic stewardship: a practical guide from the society for healthcare epidemiology of America diagnostic stewardship task force. Infect Control Hosp Epidemiol 2023;44:178–185. doi: 10.1017/ice.2023.5 36786646

[22] Yeshoua B , Bowman C , Dullea J , et al. Interventions to reduce repetitive ordering of low-value inpatient laboratory tests: a systematic review. BMJ Open Qual 2023;12:e002128. doi: 10.1136/bmjoq-2022-002128 PMC10040017 36958791

[23] Singh HK , Claeys KC , Advani SD , et al. Diagnostic stewardship to improve patient outcomes and healthcare-associated infection (HAI) metrics. Infect Control Hosp Epidemiol 45:405–411. doi: 10.1017/ice.2023.284 PMC11007360 38204365

