# Supplementary material for: Provider & nursing perspectives on the “panculture”: opportunities for innovative diagnostic stewardship interventions
Source: Antimicrob Steward Healthc Epidemiol. 2024 Nov 11;4(1):e195. doi: 10.1017/ash.2024.451 (PMC11574592; doi:10.1017/ash.2024.451)
Supplement: Gibas and Mermel supplementary material 1 — Gibas and Mermel supplementary material [file S2732494X24004510sup001.pdf]

Supplementary Figure 1: Medical Provider Survey

Fever Workup Survey: Medical Providers

1. I am a(n) (select one):

- ◯ Advanced practice provider (includes nurse practitioners & physician assistants)
- ◯ House officer (includes residents and fellows)
- ◯ Hospitalist
- ◯ Non-hospitalist staff physician

2. I work in (select one):

- ◯ Internal Medicine or an Internal Medicine subspecialty
- ◯ A surgical specialty or surgical subspecialty
- ◯ Pediatrics or a Pediatrics subspecialty
- ◯ Neurology
- ◯ Emergency Medicine

3. When you are notified that a patient is febrile, how often do you go to bedside to examine the patient in person? (select one):

- ◯ Never
- ◯ Rarely
- ◯ Often
- ◯ Always

4. How often do you order a full fever work up (or 'panculture') (meaning that you order at least blood cultures and a urinalysis/urine culture plus one or more of the following tests: chest X-ray, respiratory pathogen panel, sputum culture, or *C. difficile (C. dif)* testing) when called about a febrile patient? (select one):

- ◯ Never
- ◯ Rarely
- ◯ Often
- ◯ Always

5. Are you (select one):

- ◯ More likely to order a full fever work up (or 'panculture') if you go to the unit to examine the patient.
- ◯ More likely to order a full fever work up (or 'panculture') if you don't go to the unit to examine the patient.
- ◯ The decision to order a full fever work up (or 'panculture') doesn't depend on if I do or don't go to the unit to examine the patient.

6. I am more likely to order a full fever work up ('panculture') during the (select one):

- ◯ Day shift
- ◯ Night shift
- ◯ Weekend shift
- ◯ The shift does not impact my decision to order a full fever work up ('panculture').

7. When assuming care of a patient how often do you receive a written handoff for that patient?

- ◯ Never
- ◯ Rarely
- ◯ Often
- ◯ Always

8. If the handoff contains instructions to order a full fever work up (or 'panculture'), how often do you follow these instructions and order a full fever work up ('panculture') regardless of the patient's symptoms or clinical status?

- ◯ Never
- ◯ Rarely
- ◯ Often
- ◯ Always

9. How often do you feel that the handoffs/sign-outs you receive are accurate and up to date?

- ◯ Never
- ◯ Rarely
- ◯ Often
- ◯ Always

10. When you order a full fever work up (or 'panculture'), what tests do you generally order (select all that apply)?

- ☐ Blood culture
- ☐ Urine culture
- ☐ C. diff testing
- ☐ Sputum culture
- ☐ Chest X-ray
- ☐ Respiratory pathogen panel
