# Supplementary material for: Provider & nursing perspectives on the “panculture”: opportunities for innovative diagnostic stewardship interventions
Source: Antimicrob Steward Healthc Epidemiol. 2024 Nov 11;4(1):e195. doi: 10.1017/ash.2024.451 (PMC11574592; doi:10.1017/ash.2024.451)
Supplement: Gibas and Mermel supplementary material 2 — Gibas and Mermel supplementary material [file S2732494X24004510sup002.pdf]

Supplementary Figure 2: Nursing Staff Survey

Fever Workup Survey: Nursing Staff

1. I am a:

- ◯ Registered Nurse (RN)
- ◯ Licensed Practical Nurse (LPN)
- ◯ Certified nursing assistant (CNA)

2. I work in/on a(n) (select one):

- ◯ Adult intensive care unit (ICU)
- ◯ Adult stepdown unit/floor
- ◯ Adult medical-surgical (med-surg) unit/floor
- ◯ Pediatric intensive care unit (ICU)
- ◯ Pediatric stepdown unit/floor
- ◯ Pediatric medical-surgical (med-surg) unit/floor
- ◯ Oncology or stem cell transplant unit/floor
- ◯ Other type of inpatient unit/floor or Emergency Department

3. I most often work the following shift(s):

- ◯ Day shift
- ◯ Second shift
- ◯ Night/overnight shift

4. When a patient you are caring for is febrile, how often do you notify the provider on call?

- ◯ Never
- ◯ Rarely
- ◯ Often
- ◯ Always

5. When you notify a provider that a patient is febrile, how often is a full fever work up (or 'panculture') ordered (meaning that the provider orders at least blood cultures and a urinalysis/urine culture plus one or more of the following tests: chest X-ray, respiratory pathogen panel, sputum culture, or *C. difficile (C. dif)* testing?

- ◯ Never
- ◯ Rarely
- ◯ Often
- ◯ Always

6. When you notify a provider that a patient is febrile, how often does the provider come to physically evaluate the patient in person at the bedside?

- ◯ Never
- ◯ Rarely
- ◯ Often
- ◯ Always

7. When a provider orders a full fever work up (or 'panculture'), how often does the provider come to evaluate the patient in person at the bedside?

- ◯ Never
- ◯ Rarely
- ◯ Often
- ◯ Always

8. A provider is most likely to order a full fever work up (or 'panculture') for a febrile patient during the following shift:

- ◯ Day shift
- ◯ Second shift
- ◯ Night shift
- ◯ Weekend shift

9. A provider is most likely to evaluate a febrile patient at the bedside during the following shift:

- ◯ Day shift
- ◯ Second shift
- ◯ Night shift
- ◯ Weekend shift
- ◯ The shift does not impact the likelihood of a provider evaluating a patient at the bedside.

10. When a provider orders tests to work up a febrile patient, what tests do they generally order (select all that apply)?

- ☐ Blood culture
- ☐ Urine culture
- ☐ C. diff testing
- ☐ Sputum culture
- ☐ Chest X-ray
- ☐ Respiratory pathogen panel
